# Sodium Alginate/Modified Bentonite Composite Bead Adsorptive Removal of Norfloxacin: Static and Dynamic Adsorption

**DOI:** 10.3390/polym14193984

**Published:** 2022-09-23

**Authors:** Jun Zhou, Qianyu Sun

**Affiliations:** College of Urban Construction, Nanjing Tech University, Nanjing 211816, China

**Keywords:** modified bentonite granule, adsorption, sodium alginate, norfloxacin, dynamic adsorption

## Abstract

The low-cost calcium-based bentonite modified with anionic and cationic surfactants was granulated by cross-linking to sodium alginate (SA) to promote the adsorption efficiencies of norfloxacin (NOR). The characterization studies illustrated that the intercalation of cetyltrimethylammonium bromide (CTAB) and sodium dodecyl benzene sulfonate (SDBS) was successful. The modification improved the pore structure and the granular SA/organically modified bentonite composite (GOMBt) exhibited a lamellar structure with some roughness. The adsorption kinetics and isotherms indicated that adsorption of NOR on GOMBt was an endothermic process. The effects of various factors on the adsorption of NOR suggested that the maximum adsorption capacity was obtained under acidic conditions and cations improved the adsorption process. A fixed-bed column was employed to investigate the dynamic adsorption characteristics of NOR by GOMBt. The breakthrough time and bed height had a positive correlation; however, the relation of flow rate, pH, and breakthrough time had a negative correlation. The results showed that the dynamic adsorption data of NOR on GOMBt fitted Thomas and Yoon–Nelson models. The internal and external diffusion in GOMBt dynamic adsorption was not a rate-limiting step.

## 1. Introduction

Norfloxacin (NOR), a synthetic fluoroquinolone antibiotic, is widely used in human medical, livestock, and aquaculture attributed to its high antibacterial activity, good oral absorption, and low side-effects [1]. Approximately 40~90% of NOR is excreted through feces and discharged to the environment via domestic sewage, farming wastewater, and disposal of expired pharmaceuticals due to the low metabolism in humans and animals [2]. In recent years, NOR has been frequently detected in aqueous environments, and soil and other sedimentary environments [3]. Trace NOR in the environment may lead to the development of antibiotic-resistant bacteria and genes [4], which has a negative effect on aquatic organisms and ecosystems [5]. The presence of NOR in the environment is also a serious threat to human health [6]. However, traditional water and wastewater treatment technologies are insufficient to remove NOR from the aquatic environment owing to its antimicrobial activity [7]. As such, having regard to the wide spread and threats of NOR contamination in the environment, there is an increasing demand for developing an efficient and practical method to complete elimination of NOR in waters.

Currently, many techniques have been applied to treat fluoroquinolone-containing wastewater, including chemical precipitation, photolysis, ion exchange, coagulation and flocculation, complexation, ozonation, advanced oxidation processes, membrane filtration, biological remediation, and activated carbon adsorption [8,9,10,11]. With the development of the technologies, some of these technologies have low costs and relatively high removal efficiency [12]. However, these methods suffered from the disadvantages of potential toxic degradation products and long treatment time [13,14]. Among these, the adsorption technique had become a favored approach with advantages of low cost, convenient operation, higher treatment efficiency, wide source of adsorbent materials, and wide application range [15,16]. In view of the advantages of the adsorption process and the wide distribution of NOR in aquatic environments, the adsorbent is a cost-dependent part of the adsorption of NOR. Hence, it is important to find more economical, efficient, and environment-friendly adsorbents to remove NOR. Adsorbent materials based on clay minerals, and industrial and agricultural by-products or wastes have gradually attracted attention due to the low coast.

Bentonite, a clay mineral, has the advantages of low cost and high cation exchange capacity, which is favorable for adsorption [17,18]. It also has the characteristics as with other nature clays, such as a crystalline structure and a limited variety of functional groups [19]. Bentonite is a typical 2:1 layered silicate mineral mainly composed of montmorillonite, which is much cheaper than activated carbon (a common adsorbent material) [20]. Its structure consists of two Si-O tetrahedra sandwiched by an Al-O octahedron [21], leading to good cation exchange capacity (CEC) and layer expansion capability. Introducing different types of inorganic cations, organic cations or neutral molecules in the interlayer can improve the specific surface area, charge properties, and reactivity of bentonite [22]. Therefore, it can improve the adsorption performance and stability of bentonite, and synthesize various adsorbents through various modification methods, indicating its high selectivity with different contaminants. Bentonite clay has a good performance in organic matter, heavy metals, and trace pollutants removal. However, it is difficult to separate solid and liquid in the actual treatment process and is not easy to recover, which limits its application. Granulation of bentonite clay can solve the problem and make it easy to separate from an aqueous environment. Sodium alginate (SA), a natural biopolymer, has been widely used as a gelling agent and binder for preparing various composite materials for adsorption due to their rich functional groups, nontoxicity, and environmentally friendly properties. SA forms an interconnected open pore network by cross-linking with calcium ions. It can be easily used to form composite beads by encapsulating other adsorbents. Encapsulation of organically modified bentonite by SA can be used to remove NOR from wastewaters, because organically modified bentonites improve the removal of NOR, and encapsulation improves the quality of separation of the adsorbent-adsorbate.

In this study, the removal of the antibiotic NOR by adsorption using as an alternative adsorbent GOMBt (a granular SA/organically modified bentonite composite) was investigated. A novel adsorbent was prepared using organically modified bentonite as a raw material, sodium alginate (SA) as a granular carrier, and calcium chloride as a crosslinking agent, which is easy to separate from water. Adsorption of NOR was researched in static and dynamic systems. The kinetics, equilibrium, and thermodynamics of adsorption were investigated. Characterization analyses of scanning electron microscopy (SEM), X-ray diffractometry (XRD), Fourier transform infrared (FTIR) spectrometry, and N_2_ physisorption (Brunauer–Emmett–Teller (BET)) were performed with samples of organically modified bentonite granules. The effect of ionic strength and pH was investigated in a static system. A fix-bed system was employed to evaluate the effect of flow rate, bed height, and pH on NOR adsorption in a continuous system. The models of Thomas, Yoon–Nelson, and Bed Depth Service Time (BDST) were employed to fit the breakthrough curves. These help to understand the dynamic adsorption process of NOR by the novel granular adsorbent and provide basic data and a theoretical basis for the utilization of the prepared organic modified bentonite granule adsorbent in continuous systems.

## 2. Materials and Methods

### 2.1. Material Acquisition

Calcium bentonite clay (Mt) was purchased from Tianjin Guangfu Fine Chemical Research Institute. The montmorillonite content is higher than 80%. The properties of Mt are shown in Table 1. Sodium hydroxide, cetyltrimethylammonium bromide (CTAB), sodium dodecyl benzene sulfonate (SDBS), anhydrous ethanol, sodium alginate (SA), calcium chloride, and polyethylene glycol 6000 (PEG) were all analytically pure, purchased from Sinopharm Group Chemical Reagent Co., Ltd (Shanghai, China). Concentrated hydrochloric acid, ammonium hydroxide, concentrated sulfuric acid, potassium chloride, and calcium chloride were all of analytical grade, purchased from Nanjing Chemical Reagent Co., Ltd. (Nanjing, China) Norfloxacin standard was purchased from the National Institute for Food and Drug Control.

### 2.2. Adsorbent Preparation

An amount of 50 g of Mt was weighed and put into a 1 L beaker. A volume of 500 mL of deionized water was added to the beaker. The mixture was stirred on a magnetic stirrer at 800 rpm for 1 h and then left for layering. The supernatant and bottom residue were discarded, and the middle bentonite slurry was collected. The slurry was dried at 105 ℃ and grinded through a 200-mesh sieve for standby.

The organically modified Mt (OMBt) was prepared as follows: A small amount of deionized water was added to 10 g of Bt and stirred. A 50 mL solution containing CTAB (110% CEC) and SDBS (20% CEC) was added to the Bt-containing suspension. The mixture was stirred on a magnetic stirrer for 2 h. After centrifugation, the supernatant was discarded, and the bottom residue was fully washed until there was no Br^−^ in the supernatant. The residue was dried at 105 °C and grinded through a 200-mesh sieve for later use.

For preparation of a granular SA/modified bentonite composite (GOMBt), SA (w(Bt) = 6%) was dissolved in deionized water. A total of 5 g of Bt or OMBt was added to SA solution and the mixture was stirred for 2 h to make Bt/OMBt mixed with SA evenly. Then, pore former PEG (w(SA) = 25%) was added and stirred for 2 h. The solid–liquid ratio was guaranteed to be about 1:7. A syringe was used to evenly drop the mixed viscous material into CaCl_2_ solution (w = 3%), and it was let to stand for 12 h for gelation reaction. After the gelation reaction, it was fully washed with deionized water and dried at 105 °C for hours. After modification and granulation, the granular bentonite (GBt) and modified bentonite granule (GOMBt) adsorbents with a diameter of 1~2 mm were obtained.

### 2.3. Characterization

Surface morphology of the samples was characterized using SEM (ZEISS Merlin, Zeiss AG, Oberkochen, Germany) equipped with energy-dispersive X-ray fluorescence spectroscopy (EDS) for analyzing surface elements. XRD (D8 Advance, Bruker, Berlin, Germany) was performed to analyze the crystal morphology of the sample surface. FTIR (Nicolet 510P, Nicolet, Madison, WI, USA) was carried out to characterize the functional groups of the sample. The sample’s specific surface area was measured according to BET (ASAP-2020, Micromeritics, Norcross, GA, USA). NOR was detected on a UV–vis spectrometer at 277 nm.

### 2.4. Static Adsorportion Experiment

#### 2.4.1. Adsorption Kinetic, Isotherm, and Thermodynamic

Adsorption kinetics of NOR on GOMBt was determined by adding 0.1 g of each adsorbent to a 50 mL stoppered conical flask containing 25 mL of NOR solution at 25 °C in an incubator shaker with a shaking speed of 150 rpm. The initial concentrations of NOR were 5 mg/L. Samples were collected at 0, 0.5, 1, 2, 4, 7, 13, 19, 24, and 30 h after filtering a 0.45 µm pore-size microporous membrane, and then the NOR concentration was tested. The results are fitted as Equations (1) and (2) [23].

(1) $$\mathrm{Pseudo}-\mathrm{first}-\mathrm{order}\;\mathrm{kinetic}\;\mathrm{model}:\;\ln\left(q_{e}-q_{t}\right)=\ln q_{e}-k_{1}t$$

(2) $$\mathrm{Pseudo}-\mathrm{second}-\mathrm{order}\;\mathrm{kinetic}\;\mathrm{model}:\;\frac{t}{q_{t}}=\frac{1}{k_{2}q_{e}{}^{2}}+\frac{t}{q_{e}}$$

where *q_e_* (mg/g) is the equilibrium adsorption capacity, *q_t_* (mg/g) is the adsorption capacity at time *t* (h), *k*_1_ (h^−1^) is the adsorption rate constant for pseudo-first-order adsorption, and *k*_2_ (g/(mg h)) is the pseudo-second-order adsorption rate constant.

Adsorption isotherms of NOR on GOMBt and GBt were carried out by adding 0.08 g of each adsorbent to a 50 mL stoppered conical flask containing 25 mL of NOR solution in an incubator shaker with a shaking speed of 150 rpm. The concentration of NOR solution varied from 2 to 20 mg/L. The experiment was performed at 298, 308, and 318 K. The samples were collected at 24 h and the final NOR concentration was tested after filtering a 0.45 µm pore-size microporous membrane. The results were fitted to Freundlich and Langmuir models as Equations (3) and (4).

(3) $$\mathrm{Langmuir}:\;q_{e}=\frac{k_{l}q_{m}C_{e}}{1+k_{l}C_{e}}$$

(4) $$\mathrm{Freundlich}:\;q_{e}=k_{f}C_{e}{}^{\frac{1}{n}}$$

where *q*_e_ (mg/g) is the equilibrium adsorption capacity of the adsorbent, *q_m_* (mg/g) is the maximum adsorption capacity of the adsorbent, *C_e_* (mg/L) is the concentration of NOR at equilibrium, *kl* (L/mg) is the Langmuir constant, *kf* (mg/g) is the Freundlich constant, and *n* is a constant evaluating the system heterogeneity.

The thermodynamic study determined the thermodynamic parameters using Equations (5) and (6).

(5) ΔG=−RTlnK

(6) $$\ln K=\frac{\Delta S}{R}-\frac{\Delta H}{RT}$$

where *R* is the universal gas constant (8.314 J/(mol·K)), *K* is the thermodynamic equilibrium constant (L/mol), *q_e_* (mg/g) is the equilibrium adsorption capacity, *C_e_* (mg/L) is the equilibrium solution phase concentration, Δ*G* (kJ/mol) is the change in Gibbs free energy, Δ*H* (kJ/mol) is the change in total enthalpy, and Δ*S* (J/(K·mol)) is the change in entropy of the process.

#### 2.4.2. Effects of pH Ionic Strength

Generally, for investigation of the effect of pH and ionic strength, the batch adsorption experiments were conducted by adding 0.1 g of each adsorbent in a stoppered conical flask, and mixing 25 mL of NOR solution at 25 °C in an incubator shaker with a shaking speed of 150 rpm. At the end of shaking, mixtures were filtered through a 0.45 µm pore-size microporous membrane and the final concentration of NOR was detected. The effect of pH was conducted by ranging the solution pH from 2 to 13. The effect of ionic strength was carried out at pH = 5, with 0, 0.01, and 0.1 M ionic strengths (NaCl, CaCl_2_). The point of zero charge (pH_PZC_) of the adsorbents was determined according to the method described in the literature [24,25].

### 2.5. Fixed-Bed-Column Adsorption Experiment

The fixed-bed adsorption of NOR was conducted in a polytetrafluoroethylene (PTFE) column (20 cm high and 1.5 cm in diameter). The column was filled with a certain amount of adsorbent material according to the experimental requirement. An appropriate amount of core sand was placed in the lower end of the column avoiding the loss of adsorbent. GOMBt was soaked with deionized water before the experiment. NOR solution was added from the top of the column through a peristaltic pump to collect the effluent regularly. The concentration of effluent was measured after filtering a 0.45 µm pore-size microporous membrane.

First, a dynamic adsorption was conducted at flow rates of 0.88, 1.33, and 2.65 mL/min with a constant inlet NOR concentration in C_0_ = 2 mg/L. The bed height, initial pH, and temperature were 15 cm, 3, and 25 °C. After obtaining the best flow condition, the effect of bed height was evaluated at a bed height of 5, 10, and 15 cm. The effect of pH was performed at pH values of 3, 5, and 7 with a bed height of 15 cm. In this study, 5% C_0_ was set as the breakthrough point and 95% C_0_ was set as the saturation point.

The adsorption capacities and removal efficiencies of exhaustion (*q_e_* and R%) were determined by Equations (7) and (8).

(7) $$q_{e}=\frac{Q}{1000m}\int_{0}^{t_{e}}\left(C_{0}-C_{t}\right)dt$$

(8) $$R\%=\left(\frac{q_{e}m}{C_{0}Qt_{e}}\right)\times 100$$

where *q_e_* (mg/g) is the saturated adsorption capacity, *m* (g) is the mass of the adsorbent, *t_e_* (min) is the saturation time, *C_t_* (mg/L) is the outlet NOR concentration, and *Q* (mL/min) is the flow rate.

### 2.6. Adsorption Model Fitting

In order to understand the dynamic adsorption process of NOR by GOMBt, mathematical models were applied to fit the breakthrough curves.

#### 2.6.1. Thomas Model

The Thomas model assumes that the external and internal diffusion resistances in the adsorption column are insignificant and that the adsorption process follows a second-order reversible reaction kinetics and the Langmuir isotherm model. The Thomas model is expressed by Equation (9).

(9) $$\frac{C_{t}}{C_{0}}=\frac{1}{1+\exp\left(\frac{k_{Th}q_{e}m}{Q}-k_{Th}C_{0}t\right)}$$

where *k_Th_* (L/(mg·min)) is the Thomas adsorption rate constant.

#### 2.6.2. Yoon–Nelson Model

The model presumes that the probability of adsorbate adsorption and breakthrough is proportional to the decrease in the probability of each adsorbate to be adsorbed within the fixed bed. This model is concise and requires no details of the characteristics of the adsorbate, as well as adsorbent and parameters of the fixed-bed column. This model is described by Equation (10).

(10) $$\frac{C_{t}}{C_{0}}=\frac{1}{1+\exp\left[k_{YN}\left(t_{1/2}-t\right)\right]}$$

where *k_YN_* (min^−1^) is the Yoon–Nelson adsorption rate constant, and *t*_1/2_ (min) is the time required for 50% removal of NOR.

## 3. Results and Discussion

### 3.1. Characteristics of the Adsorbents

#### 3.1.1. SEM and EDS Analysis

**Figure 1:**
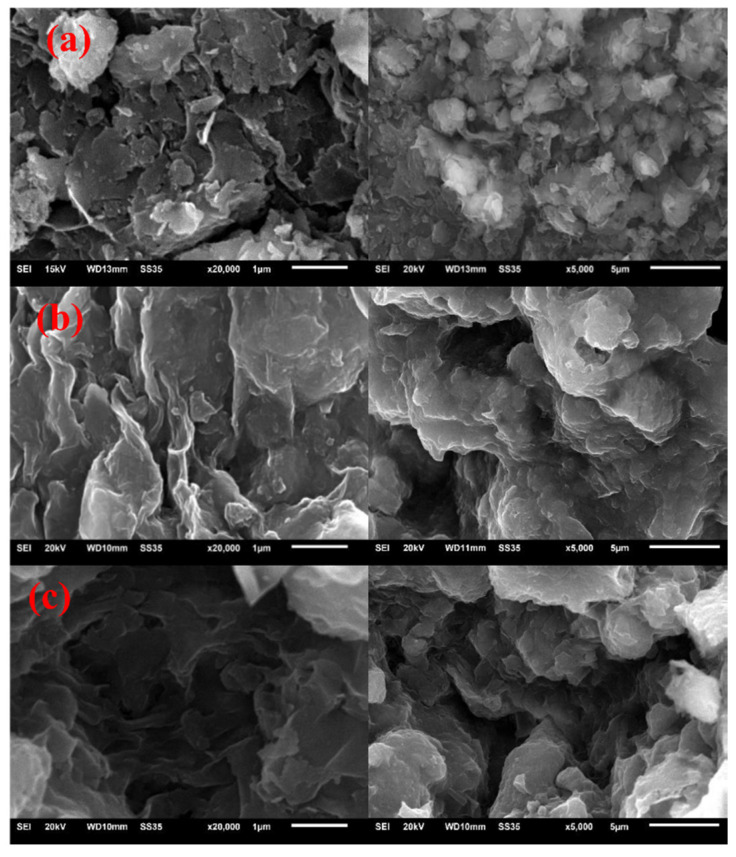
SEM of (**a**) Bt, (**b**) GBt, and (**c**) GOMBt.

The surface morphology of adsorbents is shown in Figure 1. The results showed that the surface of all absorbents was rough. Compared with Bt, the surface morphology and pores of the granulated GBt and GOMBt changed significantly. It can be seen that the layered silicate structure of bentonite did not change during the gelation process, and the layered structure of bentonite can be seen on all three samples, comprising small and thin flaky irregular aggregates in Bt. While in granulated GBt and GOMBt, the layered structure was obviously thicker and less, and the internal pores and specific surface area were significantly reduced. The interlayer spacing and internal pore volume of GOMBt were larger than those of GBt, indicating that although the embedding of SA blocked the pore structure inside the bentonite, the modification increased the layer spacing and porosity of bentonite. This improved the adsorption performance of GOMBt. The result is consistent with conclusions in other studies [26].

**Figure 2:**
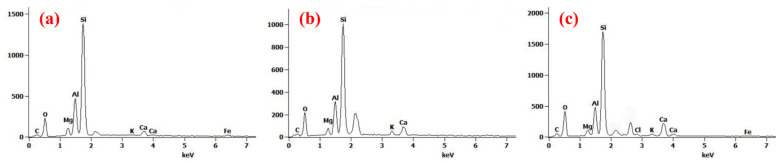
EDS spectra of (**a**) Bt, (**b**) GBt, and (**c**) GOMBt.

Table 2 and Figure 2 present the elements on the surface of adsorbents. All adsorbents showed a high percentage of O and Si. The contents of Si, Mg, Al, and Fe in GBt and GOMBt were all derived from embedded bentonite, a hydroaluminosilicate mineral. Compared with Bt, the main components of GBt and Bt were not much different, and changes in metal elements were mainly related to the replacement reaction of SA and CaCl_2_ solution. On the other hand, compared with GBt, the C and O contents of GOMBt increased significantly, while the K, Ca, and Al contents decreased. This is because part of the metal cations on the surface of bentonite are exchanged with CTAB, one end of the organic carbon chain is attached to the surface and interior of the adsorbent, and the surface hydrophobicity of the material is enhanced. This indicates that hydrophobic adsorption between GOMBt and hydrophobic organic contaminants (HOCs) such as NOR is enhanced. The detection of Cl may be due to the attachment of a small amount of Cl^−^ in CaCl_2_ solution with the positively charged carbon chains on GOMBt under electrostatic action. This suggests that CTAB was successfully inserted into bentonite under the presence of SDBS, and the modification was successful.

#### 3.1.2. XRD Analysis

**Figure 3:**
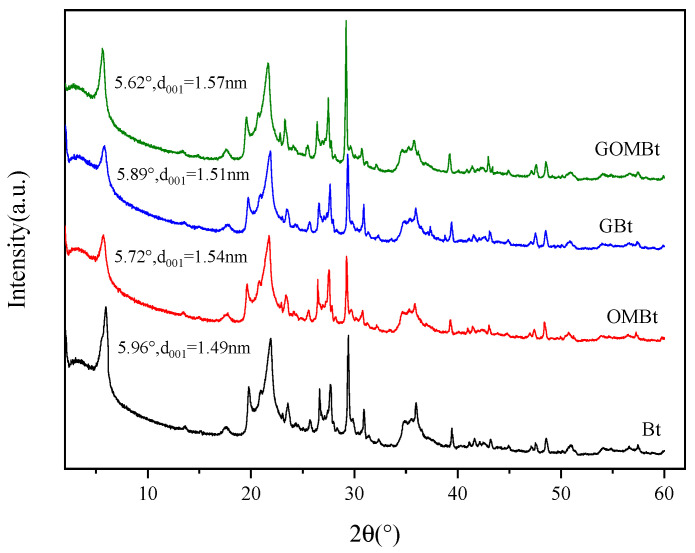
XRD patterns of adsorbents.

XRD patterns of peak positions are shown in Figure 3. The characteristic diffraction peak of bentonite (montmorillonite) on the (001) crystal plane appeared on the four samples, and the positions were at 2θ = 5.96°, 5.72°, 5.89°, and 5.62° (JCPDS No. 03-0015). The basal distances can be calculated by Bragg’s Law (2dsinθ = λ, where wavelength λ = 0.154 nm) [27]. The peak relative to the distance of the intermediate layer d_001_ was 1.49 nm for Bt clay and 1.54 nm for OMBt clay, which reduced the diffusion resistance of NOR and increased interlayer charge. The insertion of the CTAB long carbon chain increased the organic carbon content of the material and the enhanced surface hydrophobicity, improving the adsorption capacity [28]. The d_001_ values of the GBt granule and GOMBt granule were 1.51 and 1.57 nm, respectively, due to the addition of SA expanding the interlayer spacing slightly. After the granulation through encapsulation with SA, the d_001_ values of adsorbent granules were almost similar to those of the clay adsorbents. This indicated that the alginate molecules did not intercalate to silicate layers of bentonite, and the bonding between alginate and montmorillonite was only through the electrostatic interaction [29]. Some other characteristic peaks were observed in GOMBt at 2θ = 17.69°, 19.71°, 29.25 34.74°, and 35.89° belonging to montmorillonite (JCPDS No. 13-0135). The peak present at 2θ = 26.64° assigned to the (101) plane was due to the presence of quartz (JCPDS NO: 46-1045) in bentonite. Overall, the other diffraction peaks of four samples had insignificant differences, indicating that the modification of the two surfactants and the granulation did not destroy the crystal structure of bentonite.

#### 3.1.3. FTIR Analysis

**Figure 4:**
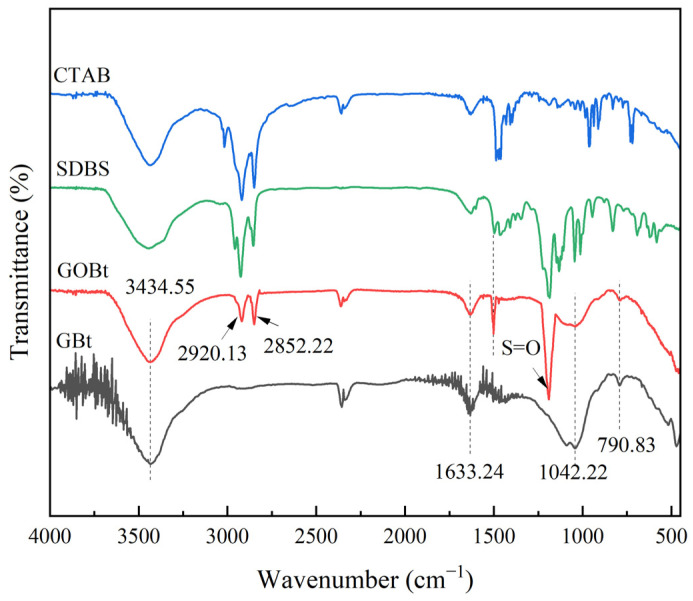
Normalized FTIR spectra of samples.

The surface functional groups of CTAB, SDBS, GBt, and GOMBt were detected by FTIR spectroscopy, which is shown in Figure 4. The characteristic absorption peaks of bentonite clay appeared at 3434.55, 1633.24, 1042.22, 790.83, and 517.25 cm^−1^ for GBt and GOMBt. A broad band centered on 3434.55 cm^−1^ was assigned to the -OH stretching vibration of H-O-H between montmorillonite layers, which was caused by the adsorption of water between the bentonite layers. The peak at 1633.24 cm^−1^ was attributed to COO^−^ bending vibration, which belongs to the presence of alginate in granules [30]. The peak at 1042.22 cm^−1^ corresponding to Si-O-Si stretching vibration in the montmorillonite lattice and the peak at 790.83 cm^−1^ assigned to Al-O-Si bending vibration between montmorillonite layers were observed on GBt and GOMBt. Compared with GBt, stretching vibration peaks of C-H in -CH_2_ and -CH_3_ were found at 2920.13 and 2852.22 cm^−1^, respectively, which were the characteristic peaks of CTAB. Meanwhile, the characteristic peak of the benzene ring at about 1500 cm^−1^ and the deformation vibration of S=O in SO_2_ at about 1189 cm^−1^, belonging to SDBS, were observed on GOMBt but not on GBt. This was ascribed to the modification. The FTIR results indicated that CTAB/SDBS compound modification was successful.

#### 3.1.4. BET Analysis

Table 3 summarizes the data obtained from BET analysis. The specific surface area and pore volume of Bt were significantly larger than those of the other two adsorbents after granulation. This suggested that the embedding of SA would block some mesopores in Bt, reducing the pore volume and specific surface area. The pore volume of GOMBt was slightly larger than that of GBt. On the one hand, it proves that the insertion of CTAB/SDBS increases the interlayer spacing of bentonite, improves the pore volume, and enhances the adsorption capacity. On the other hand, the increase in pore volume in the fixed bed results in an increase in porosity and longer breakthrough time.

**Figure 5:**
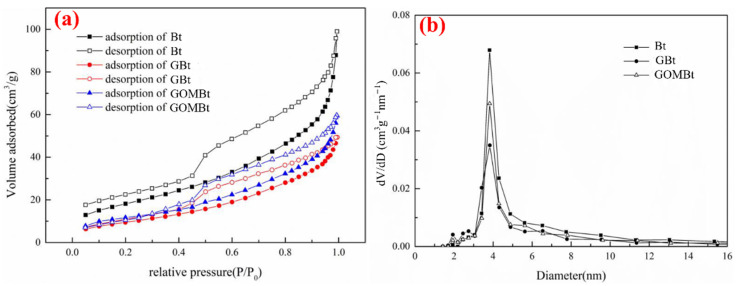
(**a**) N_2_ adsorption/desorption isotherms and (**b**) pore size distribution of Bt, GBt, and GOMBt.

As shown in Figure 5a, the adsorption capacity and adsorption rate, as well as specific surface area, of GBt and GOMBt granules decreased significantly. The adsorption isotherm of all absorbents belonged to the type III isotherm with an H3 hysteresis loop according to the IUPAC classification of adsorption isotherms, characteristic of the mesoporous adsorbent. The H3 hysteresis loop was generally found in solids consisting of aggregates of platelike particles, with a nonuniform shape or size of pores. Due to the existence of mesopores, the capillary condensation and formation of multilayers occur in the adsorption process. The samples had a weak interaction with N_2_ in the low-pressure section, indicating that a small amount of micropores existed. When P/P_0_ > 0.8, the adsorption amount of N_2_ increased rapidly, because the samples contained a certain number of mesopores and macropores, and N_2_ would accumulate in the mesopores and macropores. This is consistent with the results in Figure 5b. Slit-like pores formed by the accumulation of flaky particles existed in the absorbents according to the type of hysteresis loop. Figure 5b presents that the pore structure in the three samples is dominated by mesopores.

### 3.2. Batch Adsorption

#### 3.2.1. Adsorption Kinetics

**Figure 6:**
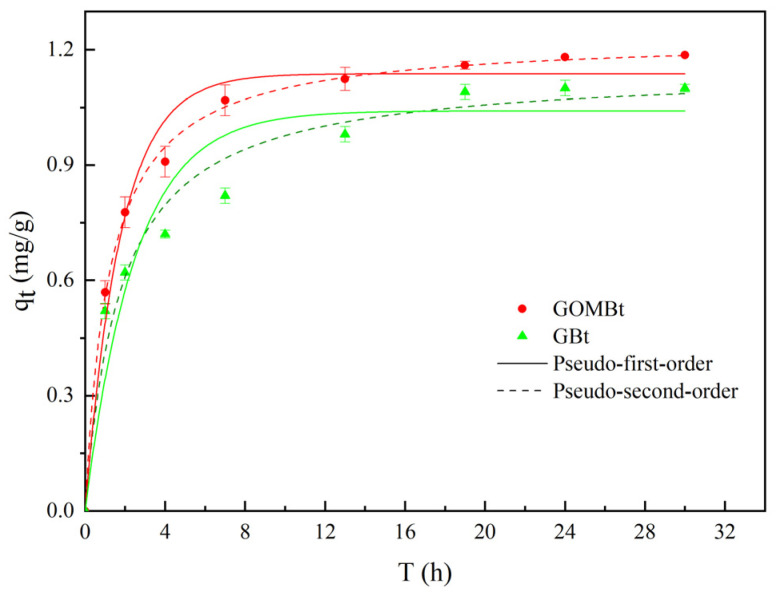
Adsorption kinetic curves of NOR on GBt and GOMBt.

The pseudo-first-order model and pseudo-second-order model were applied to simulate the NOR adsorption data. Figure 6 shows that there was a fast initial phase in the first few hours, but the adsorption rate decreased with time and it reached equilibrium within 24 h. The shape of the kinetics curves for GBt and GOMBt is similar and composed of a fast initial adsorption to the surface mesopores and macropores, followed by a slow diffusion into micropores. The process of NOR adsorption was better fitted by the pseudo-second-order kinetic model, with higher R^2^ (Table 4). The better fit by the pseudo-second-order model indicates that chemisorption is the dominant process during NOR adsorption [31] and that the final process may be slower due to diffusion into smaller pores [32]. For the pseudo-second-order model, the adsorption rate constant k of GOMBt was higher than that of GBt, which showed that GOMBt exhibited a greater NOR adsorption ability than GBt.

#### 3.2.2. Adsorption Isotherms and Thermodynamics

**Figure 7:**
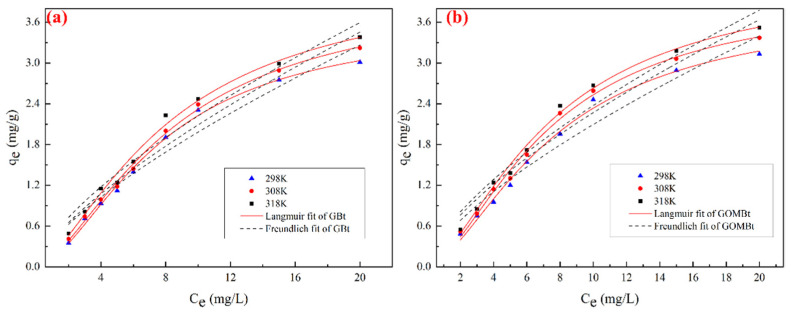
Adsorption isotherms of NOR on (**a**) GBt and (**b**) GOMBt.

The adsorption isotherms of NOR by GBt and GOMBt are shown in Figure 7 and Table 5. Langmuir and Freundlich models were applied to fit adsorption isotherms. The adsorption isotherms data were better fitted by the Langmuir model than Freundlich model due to the higher fitting coefficients (R^2^) of the Langmuir model, which demonstrated that the main adsorption type on GBt and GOMBt was monolayer adsorption. The maximum adsorption capacity of NOR by GOMBt was slightly higher than that of GBt. The maximum adsorption capacity and the kl increased with the increase in temperature, which indicated that the adsorption of NOR on GBt and GOMBt was an endothermic reaction. Higher temperature speeds up the movement rate of molecules, which is conducive to the progress of the adsorption reaction. In addition, the NOR adsorption capacity of GOMBt is greater than that of many other adsorbents reported in the literature (Table 6). Meanwhile, GOMBt is easier to separate from wastewater compared with some adsorbents possessing higher adsorption capacity. This means that GOMBt can be considered as an effective adsorbent for NOR removal.

Thermodynamic parameters (Δ*H*, Δ*G*, and Δ*S*) for NOR adsorption on C/S-M were calculated and are shown in Table 7. Positive Δ*H* indicates that the NOR adsorption on GOMBt is an endothermic process, and a reinforcement of chemisorption exists in the adsorption process of NOR on GOMBt [40]. Negative Δ*G* values suggest a spontaneous nature in the adsorption process. Meanwhile, a decrease in Δ*G* with increasing temperature indicates the higher spontaneity at higher temperature. Negative Δ*S* implies that the randomness at the GOMBt/solution interface on NOR adsorption by GOMBt is higher.

#### 3.2.3. Effect of pH and Ionic Strength

**Figure 8:**
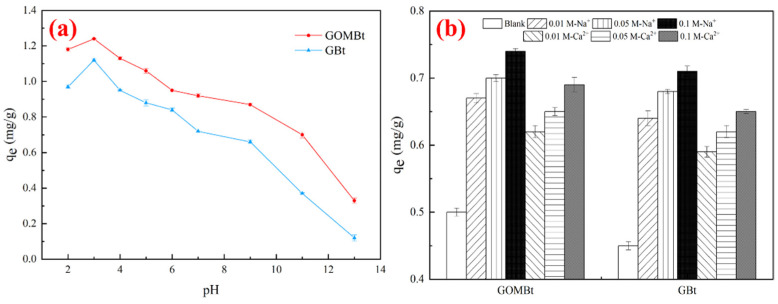
Effect of (**a**) pH and (**b**) ion strength on NOR adsorption.

The pH value reflects the nature of the physicochemical interaction of the substance in the solution and the adsorption site of the adsorbent, which in turn affects the adsorption mechanism on the surface of the solid adsorbent in the solution. When the pH increased from 2 to 13, both the adsorption capacities of NOR on GBt and GOMBt declined, while the adsorption capacity at pH 2 was lower than that at pH 3 (shown in Figure 8a). This phenomenon was attributed to NOR existing in different species at different ranges of pH [41]. NOR has two proton-binding sites (carboxyl and piperazinyl group) with pKa values of 6.34 and 8.75 [42]. NOR was in the form of NOR^+^ with a pH below 6.34 and in the form of NOR^−^ with a pH above 8.75. When the pH value was between 6.34 and 8.75, NOR existed as a zwitterionic compound. In addition, the pH_PZC_ of GBt and GOMBt were determined, which were 3.2 and 3.5, respectively. The surface of the adsorbent became negatively charged at pHs higher than pH_PZC_ and positively charged at pHs below pH_PZC_. When the pH increased from 2 to 3, the positively charged adsorbent reduced, resulting in a decline in electrostatic repulsion. Therefore, a high adsorption capacity under acidic conditions (3 < pH < 6.35) was due to the high electrostatic interactions between negatively charged absorbents and NOR^+^. Abundant H^+^ in solution might compete with NOR^+^ for adsorption sites when the pH is too low, resulting in a decrease in adsorption capacity at pH 2. When the pH was too high (pH > 8.75), the pH value promoted the decrease in NOR adsorption capacity due to the electrostatic repulsion between NOR^−^ and the negatively charged granular adsorbent surface.

Ions widely existing in industry wastewater affect the application of GOMBt. The effect of co-existing cations including Na^+^ and Ca^2+^ on the adsorption of NOR on GBt and GOMBt was also investigated with an adsorbent dosage of 0.1 g, a NOR concentration of 3 mg/L at 25 °C, and pH = 5, which is shown in Figure 8b. The addition of ions enhanced the adsorption of NOR on GBt and GOMBt. In addition, the absorption capacity increased with the cation concentration increasing from 0.01 M to 0.1 M. The reason for this phenomenon might be that the addition of cations strengthens the electrostatic attraction with the adsorbent, compresses the electric double layer, and makes the NOR molecules move from solution to GBt and GOMBt. On the other hand, the presence of ions leads to a salting out effect, which was attributed to a decrease in the solubility of NOR in water [43]. This was beneficial to diffusion of more NOR to the surface of adsorbents, resulting in an increase in adsorption capacity [44]. Moreover, cations improved the hydrophobic interaction between NOR and absorbents. Furthermore, the impact of Na^+^ on the adsorption capacity of NOR is higher than that of Ca^2+^ due to the higher affinity of hydrated ions for absorbents caused by the smaller radius [45].

### 3.3. Fixed-Bed Adsorption

#### 3.3.1. Effect of Flow Rate

**Figure 9:**
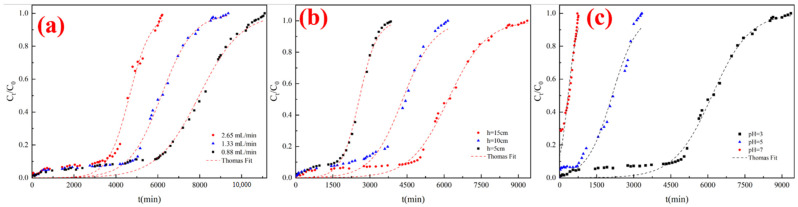
Effect of (**a**) flow rate, (**b**) bed height, and (**c**) pH on breakthrough curves.

The effect of flow rate was determined at flow rates of 0.88, 1.33, and 2.65 mL/min (corresponding empty bed contact times (EBCTs), defined as bed volume over volumetric flow rate, were 30, 20 and 10 min), an initial NOR concentration of 2 mg/L, a bed height of 15 cm, and a temperature of 25 °C. The results are illustrated in Figure 9a and Table 7. It can be found that the shape of breakthrough curves was similar with different flow rates. The breakthrough and saturation times were shorter as the flow rate increased. When the flow rate was 2.65, 1.33, and 0.88 mL/min, the breakthrough time increased from 540 min to 780 min and 1560 min, and the saturation time increased from 6060 min to 8520 min and 10,520 min. This phenomenon was related to the fact that increased flow rate provided a shorter contact time between NOR and GOMBt, leading to low interaction between NOR and GOMBt [46]. On the other hand, a high flow rate provides inadequate time for NOR membrane diffusion and intraparticle diffusion, which accelerates breakthrough and saturation [47].

#### 3.3.2. Effect of Bed Height

The breakthrough curves of NOR adsorption on GOMBt at various bed heights (5–15 cm) with a NOR concentration of 2 mg/L, EBCT of 20 min, pH of 3, and a temperature of 25 °C are shown in Figure 9b and Table 8. The result shows that the breakthrough time and saturation time evidently extended as bed height increased. The breakthrough curve was steeper with shorter bed height. The increase in bed height means more absorbent. The increase in adsorbent mass provides more adsorbent contact surface area, leading to more adsorption sites and more time to achieve complete saturation [48]. Furthermore, the increase in the mass of the adsorbent increases the adsorption capacity [49], so the breakthrough time is prolonged, the slope of the breakthrough curve decreases, and the mass transfer area becomes longer.

#### 3.3.3. Effect of pH

Solution pH impacts electrostatic interactions, further affecting breakthrough curves and adsorption capacity. It can be seen from Figure 9c and Table 7 that breakthrough time, adsorption capacity, total adsorbed amount, and removal efficiency declined significantly when the pH increased from 3 to 7. The breakthrough time decreased rapidly when the pH increased from 3 to 7, and the adsorption process reached a breakthrough point in a short time at pH = 7. This might be attributed to the forms of NOR at different pH values. When pH = 7, NOR was in the form of NOR^±^, and only a small amount of NOR^+^ underwent cation exchange with the GOMBt. The electrostatic repulsion between negatively charged GOMBt and NOR^−^ decreased the adsorption capacity and shortened the breakthrough time.

### 3.4. Modeling of Breakthrough Curves

#### 3.4.1. Fit of the Thomas Model

The Thomas model assumes that the adsorption process conforms to the Langmuir isotherm adsorption and pseudo-second-order kinetic model, which is suitable for describing the adsorption situation where internal diffusion and external diffusion resistances are extremely small. The fitting parameters of the Thomas model are shown in Table 9. The R^2^ of the fitting model were all above 0.97, indicating that the Thomas model fitted the dynamic adsorption process well. The adsorption rate constant *k_Th_* decreased with increasing bed height and decreasing flow rate and pH. This was because the decrease in flow rate increased the adsorption density, and the contact time between NOR and GOMBt was sufficient. As the bed height increased, indicating that the amount of adsorbent increased, the resistance of NOR to pass through the adsorption column increased, the breakthrough time was prolonged, and *k_Th_* decreased. The experimental values and model predictions fit well, suggesting internal and external diffusion is not a rate-limiting step for dynamic NOR adsorption on GOMBt.

#### 3.4.2. Fit of the Yoon–Nelson Model

The fit of the Yoon–Nelson model is shown in Table 10. The fit of the Yoon–Nelson model presented a high value of R^2^ (>0.97), indicating that the Yoon–Nelson model described the dynamic adsorption process well. It can be inferred from the values of *t_1/2_* in the Yoon–Nelson model that the breakthrough declined with increasing bed height and pH and decreasing flow rate. On the other hand, the changes in adsorption rate constant *k_YN_* indicated that adsorption rate increased with the rise in flow rate and pH and decreased with the reduction in bed height.

## 4. Conclusions

In this study, novel modified bentonite spherical composites (GOMBt) were synthesized by granulation of an organic modification of bentonite with CTAB and SDBS for NOR removal from aqueous solutions. GOMBt as an alternative adsorbent is efficient in removing low-concentration NOR in a static and dynamic fixed-bed system. The characterization indicated that CTAB/SDBS successfully intercalated on calcium bentonite and modification improved the adsorption capacity of GOMBt. It can be deduced from the kinetic model that the controlling step is chemisorption. The Langmuir model described the adsorption of NOR on GOMBt better. The NOR adsorption is an endothermic process. The cations were favorable to NOR adsorption and the removal efficiency was higher at pH 3. During dynamic fixed-bed adsorption, the breakthrough time decreased as the flow rate and pH increased and the breakthrough time prolonged with increasing bed height. The Thomas and Yoon–Nelson models showed acceptable adaptation to the breakthrough curves. The fit of the Thomas model indicated internal diffusion and external diffusion were not the limiting steps in the adsorption process. Accordingly, this study provided useful information for the design of a fixed-bed column for a ciprofloxacin or norfloxacin-activated carbon system. Consequently, GOMBt can be considered a promising absorbent to treat NOR-contained wastewater. This study provided significant information for NOR dynastic adsorption on GOMBt, a modified bentonite granule.

## Figures and Tables

**Table 1:** The properties of Mt.

Sample	CEC (mmol/100 g)	Ethylene Blue Adsorbed (g/100 g)	Gelling Value (mL/15 g)	Swelling Capacity (mL/g)
Mt	53.5	73.03	95	8.7

**Table 2:** EDS characterization of adsorbents.

Sample	Bt		GBt		GOMBt	
Element	Weight Percent (%)	Atomic Percent (%)	Weight Percent (%)	Atomic Percent (%)	Weight Percent (%)	Atomic Percent (%)
C K	5.89	11.81	5.33	12.20	11.46	19.96
O K	22.18	33.41	24.03	41.29	36.94	48.29
Mg K	2.34	2.32	1.21	1.85	1.38	1.19
Al K	11.91	10.63	8.07	8.43	6.52	5.06
Si K	42.6	36.55	29.17	28.55	25.22	18.78
K K	0.54	0.33	2.76	1.8	0.54	0.29
Ca K	4.20	2.53	6.01	4.12	5.37	2.8
Fe K	3.73	1.61	/	/	0.83	0.31
Cl K	/	/	/	/	4.3	2.54

**Table 3:** The BET parameter of Bt, GBt, and GOMBt.

Sample	S_BET_ (m^2^/g)	Pore Volume (cm^3^/g)	Average Pore Size (nm)
Bt	66.575	0.1532	3.821
GBt	35.657	0.07655	3.831
GOMBt	36.500	0.09753	3.825

**Table 4:** Adsorption kinetic parameters for NOR adsorption on absorbents.

Sample	Pseudo-First-Order			Pseudo-Second-Order		
	k_1_ (h^−1^)	q_e_ (mg/g)	R^2^	k_2_ (g/(mg·h))	q_e_ (mg/g)	R^2^
GOMBt	0.5613	1.137	0.977	0.6725	1.232	0.998
GBt	0.4006	1.041	0.906	0.4896	1.150	0.965

**Table 5:** Adsorption isotherm parameters for NOR adsorption on absorbents.

Sample	Temperature	Langmuir			Freundlich		
		k_l_ (L/mg)	q_m_ (mg/g)	R^2^	k_f_ (mg/g)	1/*n*	R^2^
GBt	298K	0.0328	3.627	0.995	0.379	0.7184	0.945
GBt	308K	0.0357	4.032	0.996	0.393	0.7252	0.957
GBt	318K	0.0431	4.267	0.99	0.449	0.6940	0.955
GOMBt	298K	0.0375	3.846	0.992	0.424	0.6974	0.946
GOMBt	308K	0.0397	4.043	0.994	0.474	0.6801	0.943
GOMBt	318K	0.0445	4.283	0.994	0.508	0.6700	0.949

**Table 6:** Comparisons of NOR adsorption capacity among different adsorbents.

Adsorbent	Adsorption Capacity (mg/g)	Reference
Molecular-imprinted particles (MIPs)	2.99	[33]
Modified coal fly ash	2.17	[34]
Cauliflower roots biochar	4.63	[35]
KOH-modified biochar	2.80	[36]
Hematite–biochar composites	1.68	[37]
Pomelo peel-based biochar	3.75	[38]
Iron ore waste (IOW-14)	6.48	[39]
GOMBt	3.846	This study

**Table 7:** Thermodynamics parameters for NOR adsorption on GOMBt.

T (K)	ΔH (KJ/mol)	ΔS (J/mol·k)	ΔG (KJ/mol)
298	6.74	158.02	−40.38
308			−41.88
318			−43.57

**Table 8:** Parameters in fixed-bed column for NOR adsorption by GOMBt (C_0_ = 2 mg/L).

Q (mL/min)	EBCT (min)	H (cm)	pH	t_b_ (min)	t_e_ (min)	q_e_ (mg/g)	R (%)
2.65	10	15	3	3960	6060	2.29	73.88
1.33	20	15	3	5100	8520	1.55	70.57
0.88	30	15	3	6300	10,560	1.30	72.31
0.88	20	10	3	3080	5940	1.11	78.99
0.44	20	5	3	1983	3660	0.68	72.96
1.33	20	15	5	720	3240	0.57	68.03
1.33	20	15	7	180	726	0.09	48.03

**Table 9:** The Thomas model fitting parameters under different conditions with C_0_ = 2 mg/L.

Q (mL/min)	EBCT (min)	H (cm)	pH	k_Th_ mL/(min·mg)	q_e_ (mg/g)	R^2^
2.65	10	15	3	0.98	2.38	0.980
1.33	20	15	3	0.655	1.59	0.991
0.88	30	15	3	0.4985	1.36	0.990
0.88	20	10	3	0.79	1.11	0.982
0.44	20	5	3	1.4	0.65	0.991
1.33	20	15	5	0.98	0.55	0.978
1.33	20	15	7	2.735	0.09	0.941

**Table 10:** The Yoon–Nelson model fitting parameters under different conditions with C_0_ = 2 mg/L.

Q (mL/min)	EBCT (min)	H (cm)	pH	k_YN_ × 10^−3^ (min^−1^)	t_1/2_ (min)	R^2^
2.65	10	15	3	1.96	4647.62	0.980
1.33	20	15	3	1.31	6178.35	0.991
0.88	30	15	3	0.997	7976.33	0.990
0.88	20	10	3	1.58	4334.82	0.982
0.44	20	5	3	2.8	2565.03	0.991
1.33	20	15	5	1.96	2127.21	0.978
1.33	20	15	7	5.47	345.35	0.941

## Data Availability

Data are contained within the article.
